# Control of CydB and GltA1 Expression by the SenX3 RegX3 Two Component Regulatory System of *Mycobacterium tuberculosis*

**DOI:** 10.1371/journal.pone.0021090

**Published:** 2011-06-16

**Authors:** Gretta Roberts, Indumathi S. Vadrevu, Murty V. Madiraju, Tanya Parish

**Affiliations:** 1 Queen Mary University of London, Barts and the London School of Medicine and Dentistry, London, United Kingdom; 2 Biomedical Research, The University of Texas Health Center, Tyler, Texas, United States of America; 3 Infectious Disease Research Institute, Seattle, Washington, United States of America; University of Hyderabad, India

## Abstract

Two component regulatory systems are used widely by bacteria to coordinate changes in global gene expression profiles in response to environmental signals. The SenX3-RegX3 two component system of *Mycobacterium tuberculosis* has previously been shown to play a role in virulence and phosphate-responsive control of gene expression. We demonstrate that expression of SenX3-RegX3 is controlled in response to growth conditions, although the absolute changes are small. Global gene expression profiling of a RegX3 deletion strain and wild-type strain in different culture conditions (static, microaerobic, anaerobic), as well as in an over-expressing strain identified a number of genes with changed expression patterns. Among those were genes previously identified as differentially regulated in aerobic culture, including *ald* (encoding alanine dehydrogenase) *cyd*,encoding a subunit of the cytochrome D ubiquinol oxidase, and *gltA1*, encoding a citrate synthase. Promoter activity in the upstream regions of both *cydB* and *gltA1* was altered in the RegX3 deletion strain. DNA-binding assays confirmed that RegX3 binds to the promoter regions of *ald*, *cydB* and *gltA1* in a phosphorylation-dependent manner. Taken together these data suggest a direct role for the SenX-RegX3 system in modulating expression of aerobic respiration, in addition to its role during phosphate limitation.

## Introduction

*Mycobacterium tuberculosis* is a sophisticated pathogen with a long and complex association with its human host. In contrast to many infectious agents, *M. tuberculosis* exhibits a tendency to remain latent or persistent in the target organ, the lung, for decades before reactivation leads to symptomatic disease. The capacity of this organism to establish both primary and secondary infections and to maintain its presence within the lung granuloma is predicted to be dependent on its ability to respond to changes in the environment. The recognition of external or internal conditions and their integration into global gene expression patterns is therefore an essential requirement of the bacteria in their own unique pathogenic lifestyle. Consequently, there has been a large push towards understanding how the mycobacteria regulate gene expression with a particular focus on global gene regulators.

Exposure to a specific external stimulus usually leads to a defined pattern of gene expression which is often mediated by global gene regulators. Among these, the two component regulatory systems (TCRs) are widely distributed amongst prokaryotic organisms [1], and the mycobacteria are no exception. *M. tuberculosis* has nine complete two component systems and each is predicted to control a subset of the genome, the regulon, in response to a single stimulus.

A TCR is usually composed of a sensory protein, which responds to a specific stimulus initially by auto-phosphorylation, then by phosphotransfer to the regulatory protein [1]. The phosphorylated regulator effects transcriptional control of a set of target genes (the regulon) by virtue of its role as a DNA-binding transcriptional regulator. Two component systems have been implicated in virulence in many bacteria, including *M. tuberculosis* [2]–[7].

The SenX3-RegX3 TCR of *M. tuberculosis* was originally identified by degenerate PCR [8]. SenX3 autophosphorylation and phosphotransfer to RegX3 has been demonstrated using recombinant proteins, confirming that these two proteins comprise a functional signal transduction system [9]. RegX3 binding to its own promoter, irrespective of phosphorylation state, and expression studies carried out in the surrogate host *Mycobacterium smegmatis* suggest that the system is autoregulated, although the induction ratio is low (approximately two-fold) [9]. SenX3 has a PAS domain, which commonly senses oxygen or redox states [7]. In addition, genes associated with low oxygen environments are differentially expressed in a RegX3 mutant [4]. The RegX3 system is also involved in modulating gene expression in response to phosphate availability in both *M. smegmatis* and *M. tuberculosis* [10], [11].

In this study we profiled the expression of the *senX3-regX3* operon during growth and demonstrate that small changes in expression levels are observed. Global gene expression profiles were determined in a RegX3 deletion strain grown under various conditions and in an over-expressing strain. Comparison with the wild type strain demonstrated that numerous genes were differentially expressed. We confirmed differential expression of two genes (*cydB* and *gltA1*) in the mutant strain using promoter activity assays. We also demonstrated binding of phosphorylated RegX3 to the *ald*, *cydAB* and *gltA1* promoter regions. These data indicate that RegX3 directly controls their expression and that these are *bona fide* members of the regulon.

## Materials and Methods

### Media and culture

*M. tuberculosis* H37Rv was grown in liquid Middlebrook 7H9 medium supplemented with 10% v/v OADC (Becton Dickinson) and 0.05% w/v Tween 80 and on solid Middlebrook 7H10 medium plus 10% v/v OADC. For over-expression studies using the acetamidase promoter Middlebrook 7H9 medium was supplemented with 0.2% w/v acetamide, 0.5% BSA replacing the OADC. Growth was carried out in either static cultures (10 mL of medium in a 50 mL conical tube standing) or aerated cultures (100 mL medium in a 450 cm^2^ roller bottle at 100 rpm). For the Wayne model, cultures of *M. tuberculosis* were grown in 17 mL of Dubos Tween Albumin medium (Dubos Broth Base, 10% v/v Dubos Medium Albumin (Becton Dickinson) 0.02% w/v Tween 80) in 16 mm glass tubes stirring at 100 rpm as previously described [12], [13].

### Quantitative RT-PCR (RT-qPCR)

Probes and primers were designed for RT-qPCR with molecular beacons for *sigA* (endogenous control), *senX3* and *regX3* using the software Primer Express. cDNA was synthesized from RNA using RT and random hexamer primers using AMV reverse transcriptase. PCR was carried out in a Taqman 7900 using a standard PCR master mix. For *senX3*, *regX3* and *sigA*, the primer pairs were SenX3-R (GAC TTC GAT GTC GGC GTT GT) and SenX3-F (CGA TTG TGT CGG AAG CGA TT), RegX3-F (ATC ACG CTG CCG CTC AAG) and RegX3-R (ACC CGC CCG CTG TTG), and SigA-F (CCG ATG ACG ACG AGG AGA TC) and SigA-R (GGC CTC CGA CTC GTC TTC A) respectively and the FAM-labelled probes used were SenX3-P (GCA CGC CAT AAG GTG GCG GCC), RegX3-P (GTC GAC CTG CTG GAA TAC CTG AG) and SigA-P (GAA GGA CAA GGA CTC CGG TGA TTT CG). In order to measure relative gene expression levels, standard curves for each primer-probe set were generated using genomic DNA. CT values were converted into the equivalent of ng using the standard curve. Control reactions without RT were used to confirm that there was no significant contaminating genomic DNA present. CT values for genomic DNA were converted to ng and subtracted from the plus RT values. In order to standardise the samples to ensure that equal amounts of cDNA were used each value was standardized to *sigA* to generate unit-less values. At least two independent RNA samples were assayed in triplicate for each gene.

### Analysis of promoter activity

For the RegX3 operon, the plasmid pIKL-R1 carrying the upstream predicted promoter region was used [5]. For other genes, the upstream regions carrying the putative promoters were amplified using primers aldF/aldR, cydF/cydR, and gltF/gltR, for the upstream regions of *ald*, *cydAB*, and *gltA1* respectively. Fragments were cloned into pUC18 to generate plasmids pOTTY1 (*gltA1*), pOTTY2 (*cyd*), and pOTTY3 (*ald*) respectively. Primer sequences: aldF TCC CCC GGG CAT ATG GCC TCG AAA AAG A; aldR TCC CCC GGG TGA TAG CCG AGC CCT CTC; cydF TCC CCC GGG ATC TGG CTC GTG GTG ATC G; cydR TCC CCC GGG ACG GTG GTG ATA CCG AAC T; gltF TCC CCC GGG ACG AGA TCG GTA GCC CTC TA; gltR TCC CCC GGG CAG CAC AGG ACA CCA ACA AA. The sub-cloned fragments were cloned into the unique *Sca*I site of the vector pSM128 as *Sma*I fragments. This plasmid is a mycobacteriophage L5-based integrating vector carrying a *lacZ* reporter gene and is therefore present in only one copy which is integrated into the chromosome. The plasmids were electroporated into *M. tuberculosis* and transformants selected on 20 µg/mL streptomycin. For each plasmid at least two independent transformants were assayed. Cell-free extracts were prepared from 10 ml static cultures grown in liquid medium for 14 days and assayed for beta-galactosidase activity. Protein concentrations were calculated using the BCA protein assay kit (Pierce).

### Construction of plasmids for inducible over-expression

Primers REGE2 GGT GGA TCC TCT AGA CTA GCC CTC GAG TTT GTA and REGE3 GGA TCC CAT ATG TTC ACC GGA TTT GTA GGA were used to amplify the *senX3*-*regX3* operon from genomic DNA. *Nde*I and *Xba*I restriction sites (underlined) were engineered into the primers and the operon gene was directionally cloned into pJFR19, a shuttle vector with origins of replication for *Escherichia coli* and mycobacteria, as a *Nde*I-*Bam*HI fragment to generate the plasmid pREV7 with the operon under the control of the acetamidase promoter of *M. smegmati*s [14], [15].

### Micro-arrays

RNA was isolated from the wild-type and mutant strains [16], labelled and competitively hybridised to genomic DNA as previously described [12] using the same microarrays as in our previous work [12]. Labelled cDNA was produced from total RNA by incorporation of either Cy3 or Cy5 dCTP (Amersham) using reverse transcription. Genomic DNA was labelled with either Cy3 or Cy5 dCTP using the Klenow fragment of DNA polymerase and random primers in accordance with the manufacturers protocol (Life Technologies). Labelled DNA and cDNA were mixed and purified using a Qiagen MiniElute kit and hybridised to *M. tuberculosis* whole genome PCR-product microarrays. For each condition at least three RNA samples were isolated from independent cultures. The labelled cDNA was hybridised in duplicate in competition with labelled genomic DNA (control). The dyes were swapped between the two hybridisations so that for each RNA sample, a slide with Cy3-labelled cDNA and Cy5-labelled genomic DNA was prepared and a slide with Cy5-labelled cDNA and Cy3-labelled genomic DNA was prepared. Each gene was present on the array twice, giving a total of twelve readings per gene per condition. Slides were scanned and the image acquisition programme Genepix Pro (Axon Instruments) was used to capture and analyse the fluorescent signals. Data was loaded into Genespring (Silicon Genetics) for analysis and normalised as follows. Values below 0.01 were set to 0.01. Each gene's measured intensity was divided by its control channel value in each sample; if the control channel was below 10 then 10 was used instead. If the control channel and the signal channel were both below 10 then no data was reported. Each measurement was divided by the 50th percentile of all measurements in that sample. The percentile was calculated with all raw measurements above 10, using all genes not marked absent. Culture conditions were NRP1- 7 day Wayne model; NRP2 – 14 day Wayne model; 10 mL static culture after 4 weeks Days; 100 mL roller culture after 7 days.

### Over-expression, purification and phosphorylation of recombinant proteins

The coding region of *regX3* was cloned under the control of the bacteriophage T7 promoter in pET19b and expressed in *E. coli* strain BL21(DE3) lysogen carrying pLys plasmid (Novagen). Bacterial cultures were grown to an OD_600_ of 0.6, IPTG added to a final concentration of 1 mM and incubated for 3 h. Recombinant RegX3 protein was purified under soluble conditions on Ni-NTA affinity columns (Qiagen) following the manufacturer's instructions. Peak fractions containing RegX3 were pooled and dialyzed for 4 h in Storage Buffer (25 mM HEPES pH 7.2, 0.1 mM EDTA, 10% w/v glycerol, 1 mM DTT). The purity of the RegX3 protein was monitored by SDS-PAGE followed by Coomassie brilliant blue staining. The identity of RegX3 was confirmed by MALDI-TOF MS. A recombinant plasmid expressing EnvZ as a MalE-EnvZ fusion protein was a generous gift from Dr. M. Igo (U.C. Davis, California). Soluble EnvZ was purified on amylose affinity columns following the manufacturer's instructions (NEB). Over-production and purification of MtrA protein was as described [5]. The phosphotransfer reaction was carried out in 50 mM Tris-HCl pH 7.5, 50 mM KCl, 20 mM MgCl2, 1 mM DTT, 1 µM EnvZ and 2 µM MtrA or 1 µM RegX protein. Reactions were initiated by the addition of radioactive γ-^32^P-ATP and incubated at 37°C for 15 min. Samples were removed, diluted with SDS-PAGE sample buffer and resolved in 12% w/v polyacrylamide gels under denaturing conditions.

### Electrophoretic mobility shift assays

Select promoter fragments (*cydAB*, *glt*A, *ald*) were amplified using fluorescence-labelled universal forward and reverse primers from plasmids pOTTY1, pOTTY2, and pOTTY3. All PCR products, except *glt*A were approximately 320 bp whereas *glt*A was 530 bp. PCR products were gel-purified and used in DNA-binding experiments. For competition experiments the same fragments were amplified using non-fluorescent labeled universal forward and reverse primers. DNA binding experiments were performed in 20 µl buffer containing 50 mM Tris-HCl, pH 7.0, 50 mM NaCl; 10 mM MgCl_2_; 10 mM CaCl_2_ 1 mM DTT; 5% w/v glycerol, 0.01% w/v NP-40, 0.5 µM poly dI/dC, 1 µg sheared salmon sperm DNA, 0.1 mg BSA, either 200 fmoles of FITC-labelled *glt*A or 100 fmoles of FITC-labelled *cydAB*, and *ald* DNA fragments and RegX3 protein at the indicated concentration. Reactions were incubated for 15 min prior to mixing with 5 µl of 50% w/v glycerol. Samples were resolved at 4°C in 5% polyacrylamide gels. The unlabelled promoter fragments of *cydAB*, *glt*A, and *ald*A, (cold competitor) were used at 100 fold excess as compared to FITC-labelled DNA fragments where required. Acrylamide gels were scanned using a Molecular Imager (Fx) and data were analyzed using Quantity One software (BioRad).

## Results

### Expression of the *senX3-regX3* operon during growth

We, and others, have previously demonstrated that the SenX3-RegX3 two component regulatory system is required for the full virulence of *M. tuberculosis* in both macrophage and murine infection models [5], [7]. In addition, we identified a number of genes differentially regulated in a RegX3 deletion mutant using whole genome expression profiling [5]. Recent work confirmed that RegX3 controls the expression of several genes involved in phosphate uptake in response to phosphate limitation [11], although it is not clear if the SenX3 sensor directly senses phosphate limitation, or if this is mediated via the Pst proteins [10].

**Figure 1:**
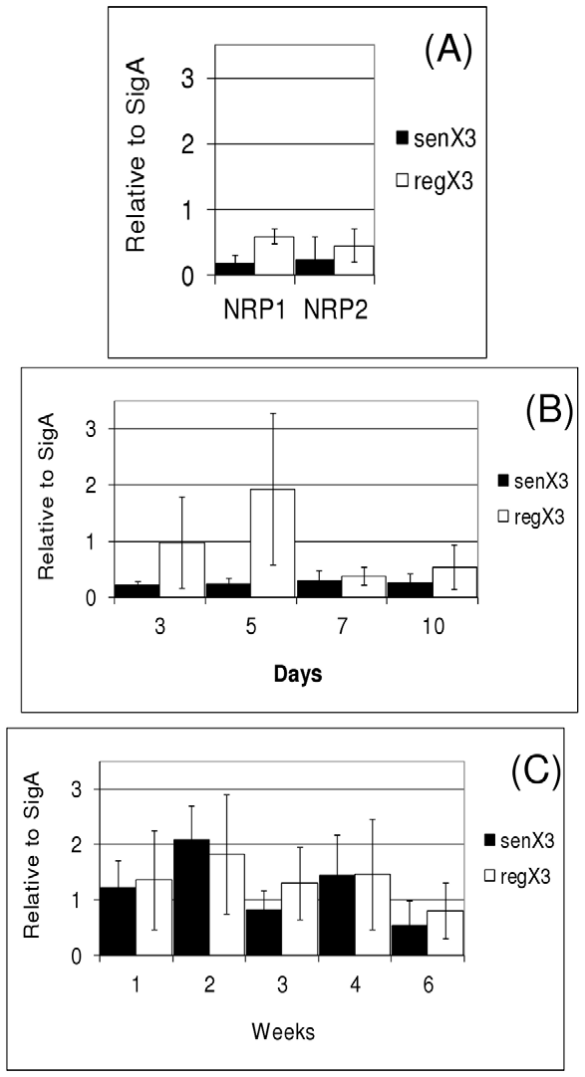
Expression of *senX3* and *regX3* at the mRNA level. Levels of mRNA were measured using RT-qPCR. The amount of mRNA is given as an arbitrary value standardized to *sigA* expression values. The mean ± standard deviation of at least two independent samples assayed in triplicate is given. (A) Wayne model cultures; NRP1 = microaerobic, NRP2 = anaerobic. (B) Roller 100 ml cultures. (C) Static 10 ml cultures.

**Figure 2:**
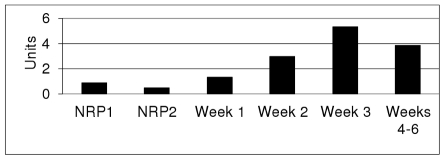
Promoter activity for the *senX3-regX3* operon under different culture conditions. P_senX3_ activity was measured in *M. tuberculosis*. Promoter activity results are the mean of a minimum of two independent cultures assayed in duplicate. Units are given in nmol O-nitro phenyl galactoside produced per min per mg total protein. Wayne model cultures; NRP1 microaerobic, NRP2 = anaerobic (days 7 and 14 respectively). Weeks 1–6 are from static cultures (10 mL).

We were interested in further profiling the expression pattern of the RegX3 operon. Our previous work had indicated that this operon is not up-regulated in response to a number of stresses, although its profile during growth was unknown [5]. We determined expression of the operon at various growth points to see if there was any growth phase-dependence. We used RT-qPCR to measure mRNA levels (Figure 1) and monitored promoter activity using a LacZ reporter (Figure 2) during growth in different culture conditions. Growth conditions were (i) highly aerobic, 100 ml roller cultures (ii) static, 10 ml aerobic cultures incubated standing with no agitation and (iii) hypoxic cultures. The hypoxic cultures were generated using the Wayne model [13]; in this system, the cultures are stirred slowly in tubes with a limited head space and a gradual depletion of oxygen occurs, resulting in the bacteria entering a non-replicating persistent (NRP) state. We used the 7 day timepoint, when the cultures are microaerobic and the cells have stopped dividing, although cell enlargement and thickening is still occurring (NRP1) [13] and the 14 day timepoint, when the cultures are anaerobic and cell division and cell enlargement has ceased (NRP2) [13].

Expression of *senX3* and *regX3* was measured at the mRNA level using RT-qPCR and standardized to *sigA*. The expression levels of both genes were low in hypoxic conditions (Figure 1A; NRP1 and NRP2), but surprisingly *regX3* expression levels were higher than *senX3* levels, (although we cannot exclude that this is due to differences in the efficiency of amplification in the RT step). There was no change in the relative levels of *senX3* or *regX3* mRNA between the microaerobic (NRP1) and anaerobic (NRP2) phases. However, during hypoxia, the mRNA content of the cell decreases [12] and the expression of most genes is reduced, and it is likely that *sigA* expression follows the same pattern. Thus although the relative level of expression did not change, this represents a real decrease in the absolute level of expression i.e. fewer mRNA molecules per cell.

In roller cultures the level of expression of *regX3* was raised at days 3 and 5, although this showed greater variation between cultures (Figure 1B). After day 7 the levels were reduced once more to the low level seen in hypoxia. This suggests that there is a transient up-regulation of the operon by up to four-fold in the early stages of aerobic growth.

In the static cultures (Figure 1C), the expression of both genes was higher than in the hypoxic cultures, although there was a larger variation in expression level. For *senX3*, expression was significantly higher in all the static cultures than in the roller and hypoxic cultures (p<0.05, Student's t-test). For *regX3*, expression in static cultures was significantly higher than the hypoxic cultures and the day 7 and 10 roller cultures (p<0.05, Student's t-test). Interestingly, in the static cultures *senX3* expression levels were similar to those of *regX3* (Figure 1C), in contrast to the roller and hypoxic cultures, where the relative levels were lower. This could result from a second promoter driving expression of *regX3* independently from *senX3*. However, we did not find any promoter activity in the intergenic region (data not shown) and RegX3 does not bind to this region [9]. It is unusual for the expression of an upstream gene in an operon to be modulated, but it is possible that other regulatory mechanisms, such as anti-termination or changes in mRNA stability are occurring.

As previously noted [5], promoter activity was generally low (Figure 2). In the static cultures, promoter activity showed a small, but progressive increase up to 3 weeks of culture and then a decrease in activity after 4 weeks as the oxygen content decreased and the cells become more anoxic. The hypoxic cultures had lower promoter activity than the static cultures.

Taken together these results suggest that there is an element of transcriptional control in response to oxygen levels and/or growth phase/rate i.e. the operon is expressed maximally at early log phase in aerobic conditions and down-regulated under non-replicating, anaerobic conditions. As compared to the *dosR* gene of *M. tuberculosis* which is induced several-fold in response to hypoxia, nitric oxide and other stimuli [17]–[19], this is a minor change in expression, although such small changes can still have significant biological consequences.

### Gene expression analysis

In order to evaluate the changes in gene expression directly related to perturbation of the SenX3-RegX3 system, we profiled the global mRNA population in a RegX3 deletion strain using microarrays. The strain used (Tame15) has the 3′ end of the *senX3* gene, the intergenic region and the *regX3* gene deleted [5]. We had previously conducted a comparison between the wild type and deletion strains in aerobic (roller) cultures only [5]. Since we had observed differences in the expression level of the operon between static cultures, and hypoxic (NRP1 and NRP2) cultures, we compared the gene expression patterns of the deletion mutant to the wild-type in each of these growth conditions. For each condition, a minimum of three independent RNA samples were hybridised in duplicate against genomic DNA.

Growth of the mutant strain was not impaired in any of the culture conditions employed (not shown), so that any changes in gene expression were not due to a change in growth rate. We had previously noted that the growth of the deletion strain was erratic in static culture i.e. sometimes a growth defect could be observed [5]. However, in these experiments we used cultures which were not growth-impaired to ensure that differences in gene expression were not due to changes in growth rate. A total of 484 genes were differentially regulated by more than 1.5-fold in static cultures, 212 in microaerobic and 130 in anaerobic cultures. (Tables S1, S2, S3). This confirmed that deletion of RegX3 has the least effect on gene expression under anaerobic conditions, where it is expressed at its lowest level, and the most effect in static cultures where it is expressed at its highest level.

Given that RegX3 expression levels were higher in aerobic and static cultures, we reasoned that these conditions should have the largest changes in gene expression directly resulting from the mutation. We therefore looked at the overlap between the two sets of data. Genes whose expression differed by more than 1.5-fold in static culture were compared with the list of differentially regulated genes from our previous data generated in aerobic culture [5]. Nine genes were differentially expressed in both aerobic and static cultures (Table 1), five were down-regulated and four were up-regulated in the RegX3 mutant.

**Table 1:** Differential expression of genes under static and aerobic conditions in a RegX3 deletion mutant.

ID	Gene name	Static	NRP1	NRP2	Aerobic*
Rv2626		0.26	1.23	0.44	2.15
Rv1996		0.29	0.82	0.35	2.14
Rv1131	*gltA1*	0.34	1.30	1.17	4.00
Rv1622	*cydB*	0.57	1.32	0.40	2.90
Rv2780	*ald*	0.66	1.28	0.63	3.19
Rv3749		1.53	0.71	1.52	2.84
Rv2428	*ahpC*	1.63	0.98	0.60	0.48
Rv1346	*fadE14*	1.79	1.70	0.95	0.38
Rv1148		1.82	1.12	1.10	0.48

Genes which were differentially expressed in the RegX3 deletion strain in static culture (10 ml standing cultures) were compared to the previous data for aerobic culture [5]. Genes which were significantly different in static culture were selected using a multiple t-test (p<0.05) with Benjamin Hochberg correction using Genespring X. Genes which were significantly changed are shown. For static, NRP1 and NRP2 cultures, the average normalized value was calculated in Genespring X and the ratio of expression (Mutant∶wild-type) is given. Static cultures were grown in 10 ml standing cultures. NRP1 and NRP2 were from 7 d (microaerobic) and 14 d (anaerobic) cultures in the Wayne model of hypoxia (see Methods).

*aerobic data taken from [5].

*CydB* and *ald* have both been associated with low oxygen environments [13], [20], [21]; both were down-regulated in the mutant strain under static conditions at the mRNA level. This is in contrast to our previous data showing up-regulation in aerobic (roller) conditions in the deletion strain [5]. The citrate synthase GltA1 and the conserved hypothetical proteins Rv1996 and Rv2626 were also down-regulated in static, but up-regulated in aerobic conditions. This indicates that RegX3 is required for full induction under static conditions and so could be a positive regulator. However under other conditions, the opposite effect was seen i.e. deletion of RegX3 led to increased expression under aerobic conditions. Similarly *ahpC*, *fadE14*, and Rv1148 were up-regulated under static conditions, but down-regulated under aerobic conditions in the RegX3 deletion strain. Only Rv3749 showed an inconsistent pattern (up-regulation in both conditions), which suggests it is not under the direct control of RegX3, and that this is an indirect effect. This could indicate that regulation involves other regulators in a complex network or that the changes in gene expression are indirect.

Analysis of the expression profile of these genes in static, microaerobic and anaerobic conditions revealed similar patterns (Table 1); of the five down-regulated genes, four showed expression ratios close to normal in NRP1. Similarly the up-regulated genes approached normal (ratio 1.0) levels during NRP1. These data suggest that the role of RegX3 during NRP1 adaptation is minimal, which correlates with the low level of expression seen for the RegX3 operon under these conditions.

We attempted to complement the deletion strain using an L5 mycobacteriophage-based integrating vector carrying a copy of the deleted gene, but the transformants were unstable and rapidly lost the plasmid (data not shown). We also tried complementation using an integrating vector lacking the L5 phage integrase gene (which can mediate plasmid excision) but this was also unstable and lost on passaging (data not shown). In our hands this is the only example of such severe plasmid instability using an L5-based vector. Since we were unable to complement the deletion, all comparisons were made to the wild type strain.

### The effect of SenX3-RegX3 over-expression

We also investigated the effect of over-expression of the *senX3-regX3* operon in the wild-type strain. A vector was constructed with the complete operon (pREV7) under the control of the acetamidase promoter [14], [15], which is induced by acetamide. We determined mRNA profiles of the operon over-expressing strain (carrying plasmid pREV7) under induced conditions (acetamide) in comparison to the control strain (carrying the parental vector pJFR19). Statistical analysis of the results showed that there were a number of differences between the two strains (Table S4). The two most highly up-regulated genes were *senX3* and *regX3* confirming that the over-expression had occurred (Table 2). Up-regulation of *senX3* was slightly higher than for *regX3*. This was as expected since *senX3* is upstream of *regX3* and is predicted to be co-transcribed and this effect has often been noted for genes arranged in operons, possibly due to the instability of mRNA and degradation occurring from the 3′ end. A number of other genes were differentially regulated, although the fold-changes were generally less than two-fold (Table S4). We were specifically interested in the genes previously identified as differentially expressed in the aerobic (roller) cultures of the deletion strain as this was the same condition used for growth of the over-expressor [14]. Five genes were common to both data sets (Table 2). Four of these genes showed consistency in their ratios in that the genes that were up-regulated in the deletion were down-regulated in the over-expressing strain and vice versa (Table 2). The fifth is a member of the PE family. These four genes are therefore very good candidates for those that are directly controlled at the transcriptional level by RegX3. A comparison of these genes with those differentially regulated in static cultures revealed that *cydB* was differentially regulated in the deletion and over-expressor strain, making it likely that this was not an indirect effect.

**Table 2:** Differential expression of genes after SenX3-RegX3 over-expression.

		Over-expression	Deletion*
Systematic	Gene name	Ratio	Ratio
Rv0430		1.75	0.49
Rv0490	*senX3*	5.65	n/a
Rv0491	*regX3*	3.27	0.4
Rv0867	*rpfA*	1.53	0.35
Rv1195	*PE*	0.51	0.3
Rv1622	*cydB*	0.7	2.9
Rv2337		0.77	3.94

Genes which were differentially regulated in both the over-expressing strain and the RegX3 deletion strain taken from [14]. Three transformants for the over-expressor were grown in liquid medium containing 0.2% acetamide to induce expression in rolled bottles for 7 d. The ratio of expression against the control strain (wild-type) is given.

### Differential activity of promoters in the *regX3* deletion mutant

We wanted to confirm differential expression of key genes between the RegX3 deletion and wild-type strains. We focussed on three genes, *ald*, *gltA1* and *cydB*, differentially regulated in the deletion strain in aerobic and static cultures, reasoning that these were the most likely to be directly regulated by RegX3. In order to confirm the microarray data, we looked at promoter activity in the wild-type and deletion strains.

**Figure 3:**
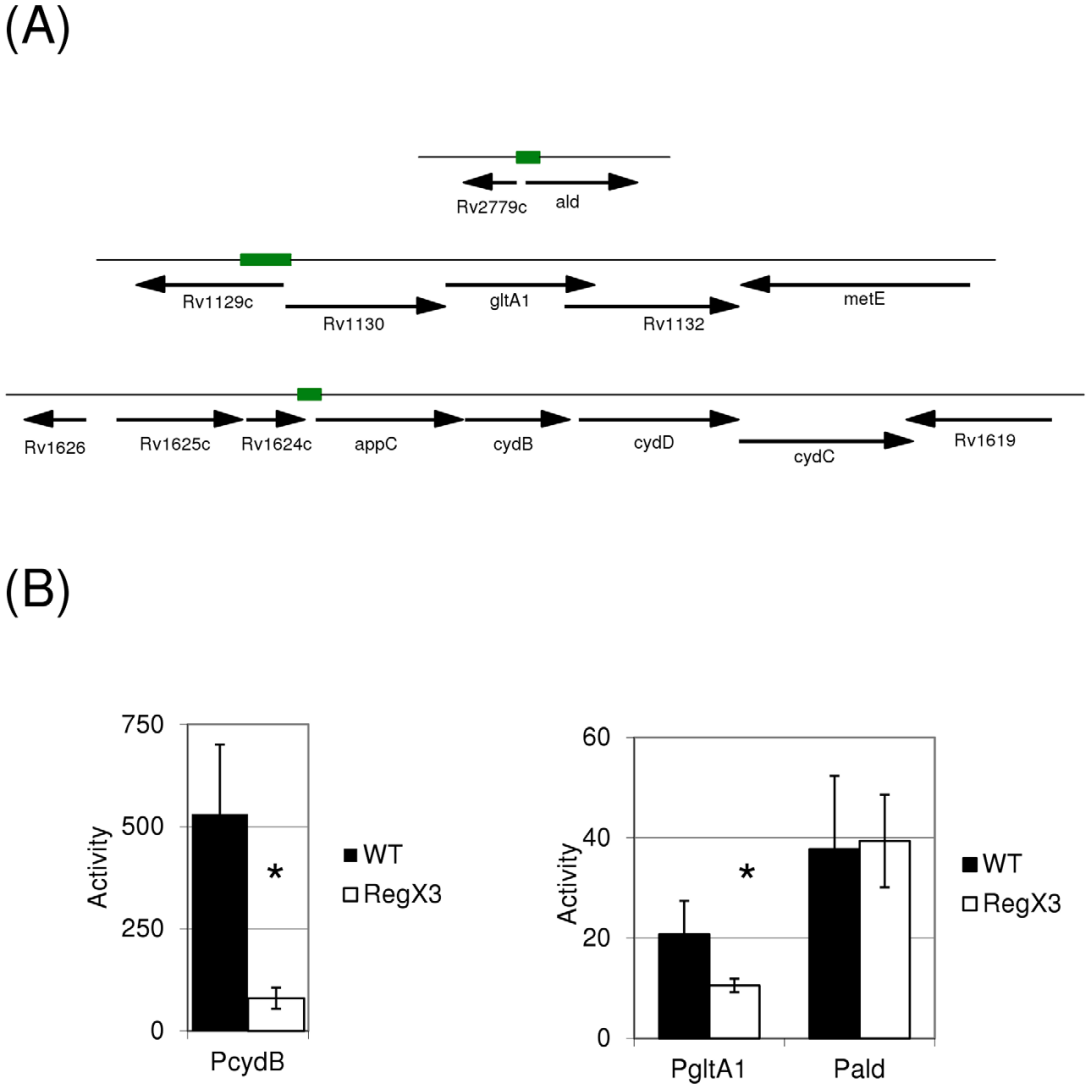
Promoter activity of selected genes in mutant and wild-type strains. (A) The chromosomal location of each gene is shown - the regions indicated as solid bars were analysed for promoter activity. (B) Promoter activity of the indicated regions was measured in wild-type and RegX3 deletion strains. Cultures were grown in 10 ml liquid medium in static cultures for 14 days. Results are the mean ± standard deviation of three independent transformant cultures conducted in duplicate (n = 6). Activity is given in nmol O-nitro phenyl galactoside produced per min per mg total protein. * indicates p<0.05 using the Student's t-test. WT – wild-type strain; RegX3 - deletion strain.

We looked at the probable operon structure of each gene to determine the most likely position of each promoter (Figure 3). *Ald* looks to be expressed independently, whereas both *cydB* and *gltA1* are in probable operons. We cloned the upstream putative promoter region of each gene/operon into the promoter probe shuttle vector pSM128 and assayed for promoter activity in the wild-type and RegX3 deletion mutant strains in static cultures.

Two of the three upstream putative promoter regions showed significantly altered activity in the mutant (p<0.05 Student's t-test) (Figure 3). The promoter for the *cydAB* operon was highly active in the wild-type strain, but activity in the mutant strain was significantly reduced (6.6-fold). P_gltA1_ was also 2-fold down-regulated in the mutant strain as compared to the wild-type. These data confirm the array data in which both *cydB* and *gltA1* were down-regulated under static conditions. In contrast to the array data, the *ald* promoter was not down-regulated under static conditions, but had the same activity in both strains.

### Phosphorylation-dependent binding of RegX3 to promoter regions

**Figure 4:**
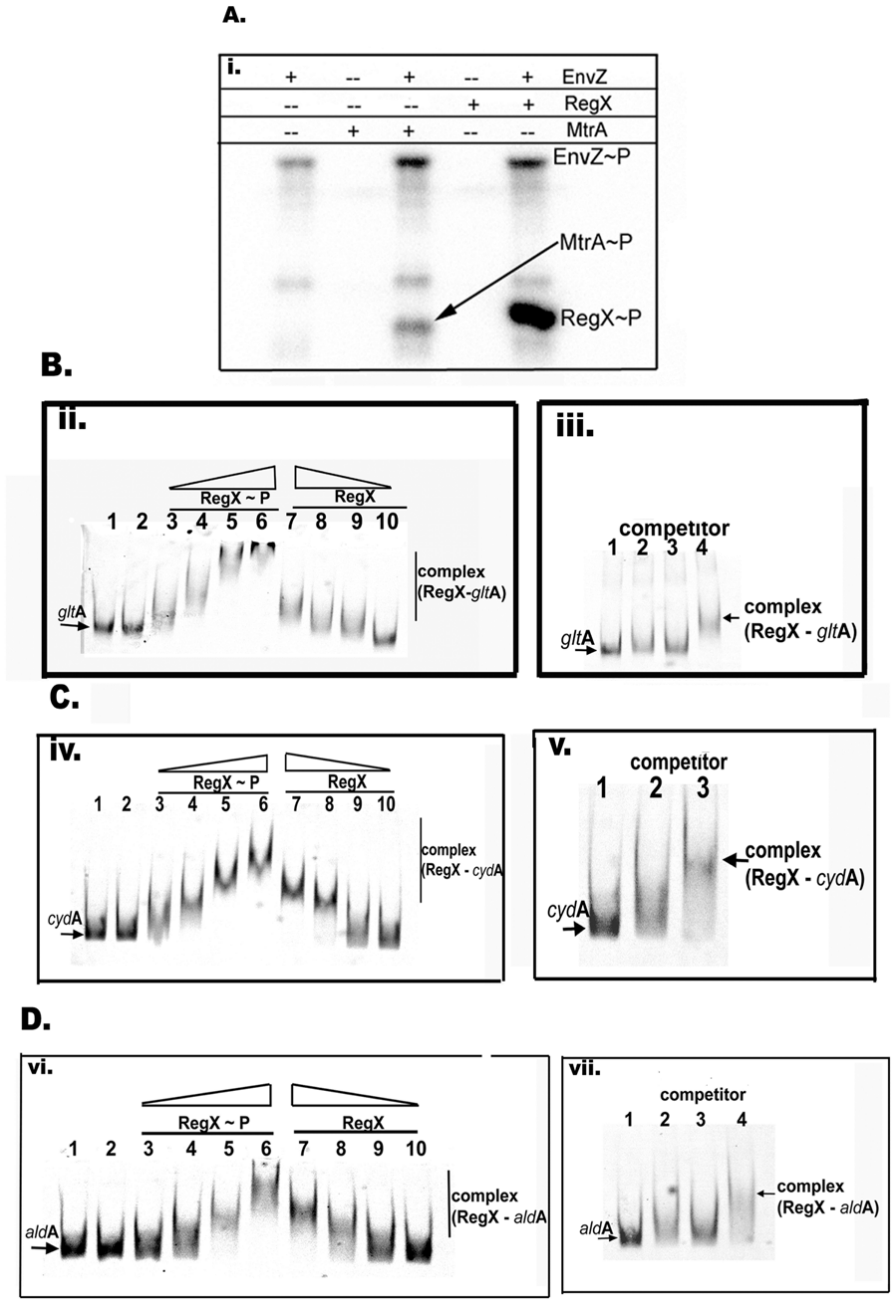
DNA binding activity of RegX3. (A) Phosphorylation of RegX3 by EnvZ. 1 µM EnvZ protein was incubated with ^32^P-ATP for 5 min. RegX3 (1 µM) or MtrA (2 µM) proteins were added and incubated for 10 min. Negative control - RegX3 or MtrA incubated with ^32^P-ATP in the absence of EnvZ. (B to E): RegX3 binding to select promoter DNA fragments. Approximately 100 fmoles of FITC-labelled *cyd*B, *ald*, or 200 fmoles of *glt*A PCR products were incubated with increasing concentrations (1, 2, 5 and 8 µM) of phosphorylated (RegX3∼P) and non-phosphorylated (RegX3) protein in DNA binding buffer (RBD) for 15 min. Protein-DNA complexes were resolved from free DNA by electrophoresis. (B-ii) P_gltA_ (C-iv) P_cydB, and_ (D-vi) P_aldA_. Lanes: 1- promoter fragment in the absence of RegX3; 2- fragments incubated with EnvZ protein alone; 3–6 lanes fragments incubated with increasing concentrations of phosphorylated RegX3, lanes 7–10 incubated with decreasing concentrations of non-phosphorylated RegX3. Panels B-iii, C-v; and D-vii refer to competition experiments carried out in the presence of 100-fold excess non-labeled promoter fragments. In these experiments RegX3 was incubated with select FITC-labeled and excess non-labeled promoter fragments for 15 min prior to electrophoresis and analysis. Lanes: 1- promoter fragments in the absence of RegX3; 2–3 in B-iii and D-vii whereas 2 in C-v RegX3 incubated in the presence of both FITC- labeled and non-labeled promoter fragments; lanes 4 in B-iii and D-vii, and 3 in C-v– RegX3 incubated in the presence of FITC-labeled promoter fragment.

The activity assays confirmed that there was differential gene expression in response to deletion of RegX3 for two of the three promoters tested. In order to confirm the direct effect of RegX3 on promoter activity, we assessed the DNA-binding activity of RegX3 to the promoters P_gltA_, P_cydAB_, and P_ald_. We expressed and purified recombinant RegX3. To evaluate the effect of phosphorylation on DNA binding activity, RegX was incubated with EnvZ, a heterologous non-specific kinase of *E. coli*. As a control, we tested the ability of EnvZ to phosphorylate MtrA [22]. As can be seen (Fig. 4A), RegX3 is phosphorylated by EnvZ, like MtrA. Using phosphorylated and non-phosphorylated RegX3 protein, we evaluated RegX3 binding to P_gltA_ (Fig. 4B), P_cydAB_ (Fig. 4C), and P_aldA_ (Fig. 4D) fragments. Our results clearly show that RegX3 binds to all three promoter fragments and that phosphorylation promoted RegX3 binding ability in all cases (Fig. 4B–D). Phosphorylation often promotes DNA-binding activity of several response regulators that have been tested, including MtrA [23]. These results indicate that, like other response regulators, RegX3 binding to its target promoters is stimulated by its phosphorylation. Incubation of phosphorylated RegX3 with FITC-labeled promoter fragments in the presence of excess non-labelled promoter fragments abolished binding (see Fig. 4, panels B-iii; C-v, and D-vii), indicating that the binding of RegX3 to these targets is specific. Together, these results confirmed that RegX3 directly involved in the regulation of the expression of *glt*A, *cydAB*, and *ald*. The upstream regions were screened for the presence of conserved motifs and for similarity to the previously-identified binding region in the RegX3 promoter [9], but there were no obvious motifs.

## Discussion

We were interested in the role of the SenX3 RegX3 two component regulatory system during growth under different oxygen tensions. Whole genome mRNA profiling and promoter assays were used to profile expression of the operon and to find genes whose expression was differentially controlled in a *regX3* deletion strain.

We looked at expression of the operon; P_senX3_ activity was dependent on growth conditions, although the fold-changes were small. However, this could still have physiological relevance, since small changes in either SenX3 or RegX3 levels could affect the amount of phosphorylated regulator and subsequently transcriptional activity of the regulon. Promoter activity did correlate with the transcriptome profiles, since the greatest effect on gene expression was seen when the promoter was activated in static cultures.

The over-expression studies were carried out after 7 d in order to provide a direct comparison to the data from our original study in which we looked at global gene expression in aerobic roller culture after 7 days in the RegX3 deletion strain. Further analysis to compare other conditions would be of benefit, for example looking at gene expression in static or anaerobic conditions after over-expression.

The down-regulation of the *cydAB* upstream region promoter activity in static culture is in contrast to our previous data showing up-regulation in aerobic roller cultures [5] suggesting that the expression of *cydAB* is changed under different oxygen tensions. Roller cultures are generally well aerated, whereas the static cultures will have lower oxygen tensions. Recent work has revealed that *cydAB* is transiently up-regulated in the mouse lung during the switch from the acute to chronic phase of infection and that a *cydC* mutant has a reduced ability to survive this transition [24]. We have previously demonstrated that the RegX3 mutant shows an attenuated phenotype in the mouse model, with an early defect in replication (up to day 30) which is recovered at later stages of infection (day 59) [5]. If RegX3 is required for the transient induction and controlled expression of *cydB*, then this could, at least partly, explain the observed phenotype. It is interesting that *cydB* (but not *cydA*) is predicted to be essential under normal growth conditions [25], [26], as it should theoretically only be required in low oxygen environments.

*CydB* was down-regulated in the over-expressor, suggesting that RegX3 is not a simple positive regulator of expression and that the regulatory system is more complex than our original hypothesis and that there may be more than one regulator of *cydAB* expression. DNA-binding of unphosphorylated RegX3 has previously been demonstrated [9], but this only occurs with its own promoter and under the conditions used here, the phosphorylated regulator preferentially bound to the target putative promoters for *ald*, *cydAB* and *gltA1*. Further work to characterize the phosphorylation state of RegX3 in various conditions and the phosphorylation/phosphatase activity of SenX3 in response to these conditions would be helpful.

Expression from P_ald_ was not significantly altered in the mutants in static culture, although changes in mRNA levels were noted and RegX binding to P_ald_ was demonstrated, suggesting that it is involved in direct regulation of the gene. *Ald* is up-regulated in stationary phase (and upon alanine supplementation) [20] suggesting that there are other regulatory elements involved; it is possible that the region cloned for the promoter analysis did not contain these elements. Further work to define the promoter and other regulatory elements under multiple conditions would answer these questions.

The SenX3 system is also involved in regulation of phosphate uptake proteins in response to phosphate concentration; under limiting conditions RegX3-dependent up-regulation of the Pst phosphate transporter system is seen [10], [11]. However, the stimulus for SenX3 remains unknown, since the sensor for phosphate depletion under these conditions is not SenX3, but a member of the Pst family. It is interesting to note that two of the genes (*gltA* and *ahpC*) identified as regulon members are up-regulated in low pH [27], suggesting that ionic concentrations may be a stimulus.

Previous work by Rickman *et al.* [7] identified a conserved PAS domain with the SenX3 protein suggesting that it may have role in sensing redox or oxygen status analogous to the ArcAB system of *E. coli*. Two of the genes that we show to be differentially regulated in the *regX3* deletion strain, namely *cydAB* and *gltA*, are controlled by ArcAB in *E. coli*. Therefore, we speculate that the role of the SenX3-RegX3 system is similar and acts to integrate the utilisation of carbon sources through the TCA cycle with defence against the potentially damaging reactive oxygen species generated by aerobic metabolism.

## Supporting Information

Table S1 **Differentially expressed genes in static culture.** Genes which were differentially expressed in the RegX3 deletion strain in static culture (10 ml standing cultures) were selected using a multiple t-test (p<0.05) with Benjamin Hochberg correction using Genespring X. Genes which were significantly changed are shown.
(DOCX)

Table S2 **Differentially expressed genes in microaerobic culture (NRP1).** Genes which were differentially expressed in the RegX3 deletion strain in microaerobic culture (NRP1) were selected using a multiple t-test (p<0.05) with Benjamin Hochberg correction using Genespring X. Genes which were significantly changed are shown.
(DOCX)

Table S3 **Differentially expressed genes in anaerobic culture (NRP2).** Genes which were differentially expressed in the RegX3 deletion strain in anaerobic culture (NRP2) were selected using a multiple t-test (p<0.05) with Benjamin Hochberg correction using Genespring X. Genes which were significantly changed are shown.
(DOCX)

Table S4 **Differentially expressed genes after SenX3-RegX3 over-expression.** Genes which were differentially expressed in the RegX3 over-expression strain in aerobic culture were selected using a multiple t-test (p<0.05) with Benjamin Hochberg correction using Genespring X. Genes which were significantly changed are shown.
(DOCX)
