# Supplementary material for: Control of CydB and GltA1 Expression by the SenX3 RegX3 Two Component Regulatory System of Mycobacterium tuberculosis
Source: PLoS One. 2011 Jun 16;6(6):e21090. doi: 10.1371/journal.pone.0021090 (PMC3116866; doi:10.1371/journal.pone.0021090)
Supplement: Table S4 — Differentially expressed genes after SenX3-RegX3 over-expression. Genes which were differentially expressed in the RegX3 over-expression strain in aerobic culture were selected using a multiple t-test (p<0.05) with Benjamin Hochberg correction using Genespring X. Genes which were significantly changed are shown. (DOCX) [file pone.0021090.s004.docx]

**Table S4.**

| \| **Up-regulated in over-expression strain** \| \| \|  \| **Down-regulated in over-expression strain** \| \| \| \| --- \| --- \| --- \| --- \| --- \| --- \| --- \| \| **Gene ID** \| **Fold change** \| **Common Name** \|  \| **Gene ID** \| **Fold change** \| **Common Name** \| \| Rv0140 \| 1.8 \|  \|  \| Rv0378 \| 2.4 \|  \| \| Rv0416 \| 1.9 \|  \|  \| Rv0786c \| 1.6 \|  \| \| Rv0430 \| 1.8 \|  \|  \| Rv0791c \| 2 \|  \| \| Rv0469 \| 1.7 \| *umaA1* \|  \| Rv1142 \| 1.6 \| *echA10* \| \| Rv0490 \| 7.4 \| *senX3* \|  \| Rv1195 \| 2.1 \| *PE* \| \| Rv0491 \| 5.1 \| *regX3* \|  \| Rv1196 \| 4.9 \| *PPE* \| \| Rv0492c \| 2.2 \|  \|  \| Rv1256c \| 1.6 \|  \| \| Rv0689c \| 1.9 \|  \|  \| Rv1344 \| 1.6 \|  \| \| Rv0749 \| 1.5 \|  \|  \| Rv1361 \| 4.4 \| *PPE* \| \| Rv0806 \| 2 \| *cpsY* \|  \| Rv1503c \| 1.6 \|  \| \| Rv0844 \| 1.5 \| *narL* \|  \| Rv1512 \| 1.6 \| *epiA* \| \| Rv0867c \| 1.5 \|  \|  \| Rv1623 \| 1.8 \| *appC* \| \| Rv0929 \| 2.2 \| *pstC2* \|  \| Rv1717 \| 1.5 \|  \| \| Rv0945 \| 1.9 \|  \|  \| Rv1727 \| 1.6 \|  \| \| Rv1046c \| 1.8 \|  \|  \| Rv2170 \| 2.1 \|  \| \| Rv1169 \| 2.1 \| *PE* \|  \| Rv2197c \| 1.6 \|  \| \| Rv1221 \| 2.4 \| *sigE* \|  \| Rv2458 \| 1.9 \|  \| \| Rv1265 \| 1.5 \|  \|  \| Rv2490 \| 1.7 \| *PE PGRS* \| \| Rv1362c \| 1.6 \|  \|  \| Rv2608 \| 1.7 \| *PPE* \| \| Rv1472 \| 1.7 \| *echA12* \|  \| Rv2832 \| 1.6 \| *ugpC* \| \| Rv1491c \| 1.6 \|  \|  \| Rv3129 \| 1.6 \|  \| \| Rv1520 \| 1.7 \|  \|  \| Rv3144 \| 1.8 \| *PPE* \| \| Rv1586c \| 1.6 \|  \|  \| Rv3471c \| 1.7 \|  \| \| Rv1611 \| 1.8 \| *trpC* \|  \| Rv3477 \| 2.8 \| *PE* \| \| Rv1922 \| 1.5 \|  \|  \| Rv3478 \| 3.6 \| *PPE* \| \| Rv1979c \| 1.6 \|  \|  \| Rv3479 \| 1.6 \|  \| \| Rv1989c \| 1.7 \|  \|  \| Rv3590 \| 1.6 \| *PE PGRS* \| \| Rv2253 \| 1.7 \|  \|  \| Rv3718c \| 1.7 \|  \| \| Rv2277c \| 1.7 \|  \|  \| Rv3773c \| 1.5 \|  \| \| Rv2464c \| 1.5 \|  \|  \| Rv3841 \| 1.8 \| *bfrB* \| \| Rv2466c \| 1.5 \|  \|  \| Rv3852 \| 2 \| *hns* \| \| Rv2543 \| 1.7 \| *lppA* \|  \| Rv3891c \| 1.8 \|  \| \| Rv2564 \| 1.7 \| *glnQ* \|  \| Rv3895c \| 2.7 \|  \| \| Rv2577 \| 1.7 \|  \|  \|  \|  \|  \| \| Rv2707 \| 1.9 \|  \|  \|  \|  \|  \| \| Rv2952 \| 1.6 \|  \|  \|  \|  \|  \| \| Rv3176 \| 1.5 \| *lipS* \|  \|  \|  \|  \| \| Rv3418 \| 1.8 \| *groES* \|  \|  \|  \|  \| \| Rv3524 \| 1.6 \|  \|  \|  \|  \|  \| \| Rv3686c \| 2 \|  \|  \|  \|  \|  \| \| Rv3694c \| 1.5 \|  \|  \|  \|  \|  \| \| Rv3719 \| 1.6 \|  \|  \|  \|  \|  \| \| Rv3921c \| 1.5 \|  \|  \|  \|  \|  \| | | |  | | |
| --- | --- | --- | --- | --- | --- | --- | --- | --- | --- | --- | --- | --- | --- | --- | --- | --- | --- | --- | --- | --- | --- | --- | --- | --- | --- | --- | --- | --- | --- | --- | --- | --- | --- | --- | --- | --- | --- | --- | --- | --- | --- | --- | --- | --- | --- | --- | --- | --- | --- | --- | --- | --- | --- | --- | --- | --- | --- | --- | --- | --- | --- | --- | --- | --- | --- | --- | --- | --- | --- | --- | --- | --- | --- | --- | --- | --- | --- | --- | --- | --- | --- | --- | --- | --- | --- | --- | --- | --- | --- | --- | --- | --- | --- | --- | --- | --- | --- | --- | --- | --- | --- | --- | --- | --- | --- | --- | --- | --- | --- | --- | --- | --- | --- | --- | --- | --- | --- | --- | --- | --- | --- | --- | --- | --- | --- | --- | --- | --- | --- | --- | --- | --- | --- | --- | --- | --- | --- | --- | --- | --- | --- | --- | --- | --- | --- | --- | --- | --- | --- | --- | --- | --- | --- | --- | --- | --- | --- | --- | --- | --- | --- | --- | --- | --- | --- | --- | --- | --- | --- | --- | --- | --- | --- | --- | --- | --- | --- | --- | --- | --- | --- | --- | --- | --- | --- | --- | --- | --- | --- | --- | --- | --- | --- | --- | --- | --- | --- | --- | --- | --- | --- | --- | --- | --- | --- | --- | --- | --- | --- | --- | --- | --- | --- | --- | --- | --- | --- | --- | --- | --- | --- | --- | --- | --- | --- | --- | --- | --- | --- | --- | --- | --- | --- | --- | --- | --- | --- | --- | --- | --- | --- | --- | --- | --- | --- | --- | --- | --- | --- | --- | --- | --- | --- | --- | --- | --- | --- | --- | --- | --- | --- | --- | --- | --- | --- | --- | --- | --- | --- | --- | --- | --- | --- | --- | --- | --- | --- | --- | --- | --- | --- | --- | --- | --- | --- | --- | --- | --- | --- | --- | --- | --- | --- | --- | --- | --- | --- | --- | --- | --- | --- | --- | --- | --- | --- | --- | --- | --- | --- | --- | --- | --- | --- | --- | --- | --- | --- | --- | --- | --- |
|  |  |  |  |  |  |
|  |  |  |  |  |  |
|  |  |  |  |  |  |
|  |  |  |  |  |  |
|  |  |  |  |  |  |
|  |  |  |  |  |  |
|  |  |  |  |  |  |
|  |  |  |  |  |  |
|  |  |  |  |  |  |
|  |  |  |  |  |  |
|  |  |  |  |  |  |
|  |  |  |  |  |  |
|  |  |  |  |  |  |
|  |  |  |  |  |  |
|  |  |  |  |  |  |
|  |  |  |  |  |  |
|  |  |  |  |  |  |
|  |  |  |  |  |  |
|  |  |  |  |  |  |
|  |  |  |  |  |  |
|  |  |  |  |  |  |
|  |  |  |  |  |  |
|  |  |  |  |  |  |
|  |  |  |  |  |  |
|  |  |  |  |  |  |
|  |  |  |  |  |  |
|  |  |  |  |  |  |
|  |  |  |  |  |  |
|  |  |  |  |  |  |
|  |  |  |  |  |  |
|  |  |  |  |  |  |
|  |  |  |  |  |  |
|  |  |  |  |  |  |
|  |  |  |  |  |  |
|  |  |  |  |  |  |
|  |  |  |  |  |  |
|  |  |  |  |  |  |
|  |  |  |  |  |  |
|  |  |  |  |  |  |
|  |  |  |  |  |  |
|  |  |  |  |  |  |
|  |  |  |  |  |  |
|  |  |  |  |  |  |
|  |  |  |  |  |  |

Three transformants for the over-expressor were grown in roller bottles in liquid medium containing 0.2% acetamide to induce expression for 7d. RNA samples were isolated from these independent cultures. The labelled cDNA was hybridised in duplicate in competition with labelled genomic DNA (control). The dyes were swapped between the two hybridisations so that for each RNA sample two hybridizations were performed. Each gene was present on the array twice, giving a total of twelve readings per gene per condition. Data was loaded into Genespring (Silicon Genetics) for analysis and normalised as follows. Values below 0.01 were set to 0.01. Each gene's measured intensity was divided by its control channel value in each sample; if the control channel was below 10 then 10 was used instead. If the control channel and the signal channel were both below 10 then no data was reported. Each measurement was divided by the 50th percentile of all measurements in that sample. The percentile was calculated with all raw measurements above 10, using all genes not marked absent. Statistical analyisis using Genespring (ANOVA or t-test with multiple correction testing) was used to find genes whose expression differed between the wild-type and deletion strain; genes whose expression changed by more than 1.5-fold are given.
